# Comments on “Machine learning‑based time series models for effective CO2 emission prediction in India” by Kumari and Singh, https://doi.org/10.1007/s11356-022–21723-8

**DOI:** 10.1007/s11356-024-33939-x

**Published:** 2024-06-11

**Authors:** Ozgur Kisi, Cesar Gabriel Leite e Melo, Lars Fischer, Hanna Brandt, Lennart Heims, Alexandre Loaec, Wais Masomy, Mohammed Parvez, Jan Roehl, Christoph Külls

**Affiliations:** 1 Department of Civil Engineering, Lübeck University of Applied Sciences, 23562 Lübeck, Germany; 2 Department of Civil Engineering, Ilia State University, 0162 Tbilisi, Georgia

The discussers wish to express their gratitude to the authors for their exploration of the effectiveness of machine learning techniques in forecasting CO_2_ emissions in India. In their study (Kumari and Singh 2023), the authors utilized six models—autoregressive-integrated moving average (ARIMA), seasonal ARIMA with exogenous factors (SARIMAX), Holt-Winters (HW), random forest (RF), long short-term memory (LSTM), and linear regression (LR) and compared their performances. Their findings indicate that the LSTM model outperforms the other models in predicting CO2 emissions in India. The discussers aim to highlight some crucial concerns that the authors and future researchers should consider.

In the study of Kumari and Singh (2023), the authors have defined linear regression as a black box model throughout the entire work (see the abstract, proposed model section on page 116605, linear regression section on page 116606, Figure 2 and linear regression forecasting section on 116612). This is definitely an incorrect characterization. On the contrary, linear regression is a white box model because it provides the user with a simple equation. The authors used annual data from 1980 to 2019 covering 40 values. Data from 1980 to 2009 (30 values) were used for training of the selected models. The critical concern in the training of machine learning (ML) methods such as RF and LSTM is the avoidance of overfitting. To mitigate overfitting, it is essential to use a sufficient amount of data when tuning the parameters of ML methods (Kisi 2010, 2014; Mao et al. 2019). Employing a larger dataset contributes to the development of a better model that converges more accurately to the true results. Conversely, a small dataset may lead to overfitting, where the model fails to properly map underlying relationships, achieving good accuracy merely by chance (Schaffer 1993). As highlighted by Alwosheel et al. (2018), there exists a rule of thumb within the ANN community, recommending the use of at least 10 times the quantity of parameters for optimal results. It is important to note that this represents the minimum sample size advised, and employing even more data is crucial for generating a more reliable and robust model. In the study by Kumari and Singh (2023), the sample size used for training LSTM models, which are more complex than the ANN models, was only 30. This training data appears insufficient to prevent overfitting and adequately train the LSTM model. On the other hand, the authors say that “Therefore, we can conclude that the random forest model is not appropriate for CO2 emission forecasting.” on page 11612. The discussers do not agree on this because the main reason might be the limited number of data used for training the random forest model. The model cannot adequately learn the investigated phenomenon with only 30 data. In addition, the authors mention on page 116608 that they filled some missing values in the pre-processing stage. However, there is no other information about the missing data and process: How many data are missing? Which method did they use for filling? It seems that the available data was originally less than 30. Unfortunately, this decreases the reliability of the developed models.

The authors compared the results of the LSTM model with those of the MK (2020) and Hewamalage et al. (2021). MK (2020) also used annual data from 1965 to 2018 and a 70:30 split ratio for training and testing stages. He used 38 data for training the ANN model. In fact, the hidden node number is not mentioned in the study. Let say 3 inputs and 3 hidden nodes were used. In this case, the ANN will have 3 × 3 + 3 = 12 weights or parameters which need to be calibrated. The training data (38 annual data) appears insufficient to adequately train the ANN model. On the other hand, MK (2020) used CO_2_ emissions data from 1965 to 2018 for India. The discussers wonder why Kumari and Singh (2023) did not use the data beyond 1980. On the other hand, the authors say that they applied ANN and recurrent ANN (RNN) to their CO_2_ emissions data on page 116614. But there is no information in the study about which control parameters are applied and what methods are used for training. The information for the LSTM and RF models is also missing. In the studies involving the application of data-driven prediction models, the modeler should transparently present all details pertaining to the modeling process. This includes specifying the constraints of the algorithms used and detailing the calculation of their control parameters. Such clarity enables other researchers to easily replicate or implement the same methodology, leading to more robust conclusions about the applied method based on the results of its application (Wu et al. 2014).

**Fig. 1:**
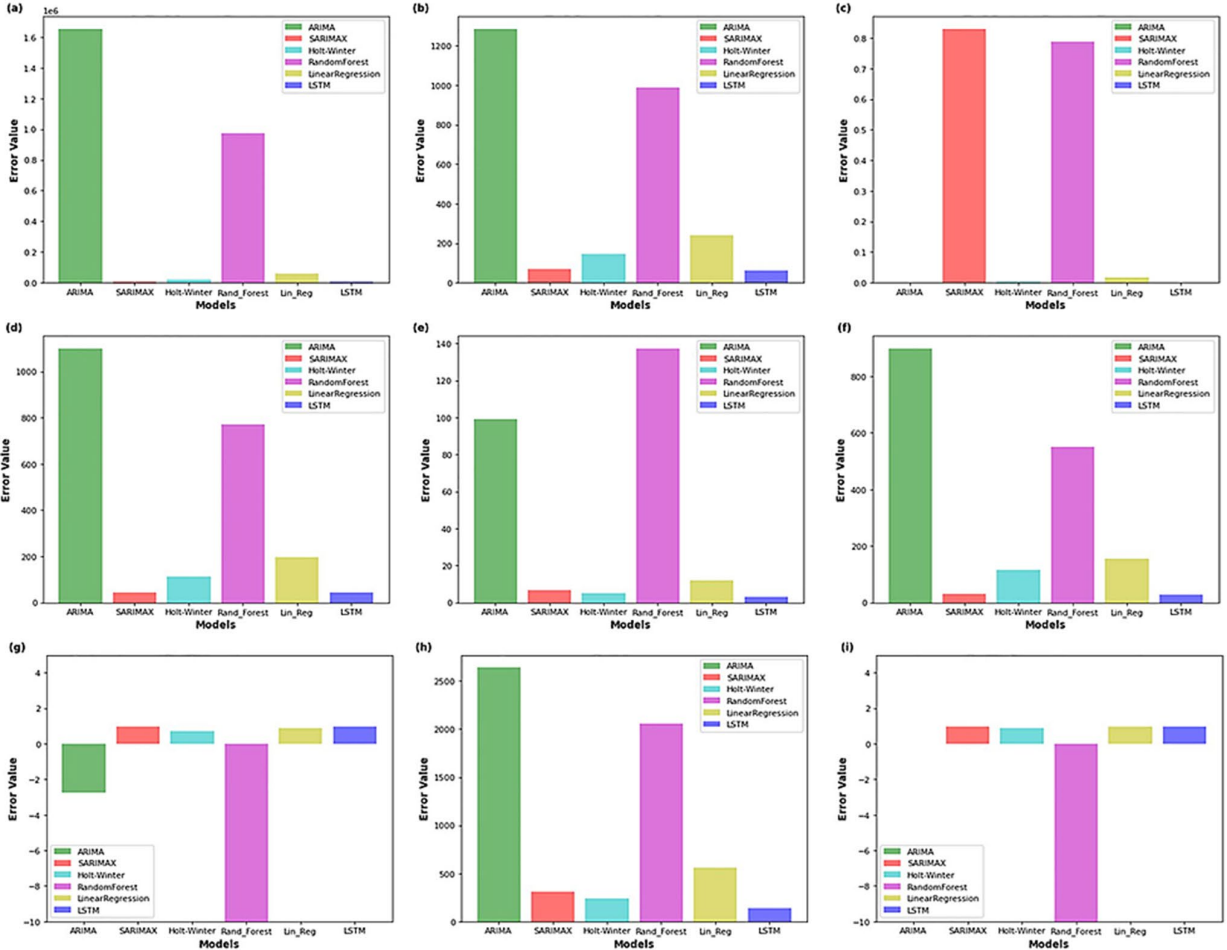
Comparison of the statistical and machine learning models in predicting CO_2_ in India (obtained from Kumari and Singh 2023)

Kumari and Singh (2023) compare the performance of the applied methods with respect to different statistics in Fig. 1. The figure is not clear and understandable. The methods names are given twice, one on the x-axis and the other as the indicators on the right upper side of the graphs, while the names of the comparison statistics are not provided. All the y-axis labels are error values. Instead of this, the exact name of the statistics could have been given. The caption of the figure also does not involve any information about the statistics used in each graph. We can understand which statistic is given in which graph after carefully reading the text. On the other hand, the selection of the graph type is not good for the comparison; it is impossible to compare the performance statistics of all the methods especially in Fig. 1a, c, and i because of different ranges. The results could have been provided in a table instead of this figure to be able to compare the methods in a more reasonable way.

Kumari and Singh (2023) provide ranges for mean absolute percentage error (MAPE) on page 116609 for defining the performance of the applied models by giving a reference to Ağbulut et al. (2021). We checked the reference and saw that they did not use MAPE statistic and they provided ranges for only relative root mean squared error. On the other hand, the thresholds or ranges of the MAPE should change depending on the investigated phenomenon. The range of “reasonable prediction” is from 20 to 50%, and it seems very large with respect to MAPE. It is not clear which stage (training or testing) Table 2 and Figure 3 report. Unnecessarily high precisions are used in the tables. For example, in Table 2, the amounts 1654252.446, 2639.0572. In Table 1, the descriptive statistics of years are provided, and this is also not reasonable at all (e.g., mean = 1999.500, minimum 1980.000, maximum 2019.000000).

The authors used three lagged values as input but did not mention how they decide this. Did they consider the limited number of training data set when deciding the input lags? The expansions of the abbreviations were not given in the text (e.g., abstract, literature review). For example, the MAPE, RMSE, and MedAE in the abstract and the RNN, LSTM-STRIPAT, MAE, and M3 in the literature review are not defined. The term “prediction” was miswritten as “predication” or “predicated” in several places (e.g., two times at the end of MAPE section on page 116609, in figures 4, 6, 7, and 8). The sentence starts with “By carefully examining …” on page 116602 (3rd paragraph) is awkward. The statement “He compared his proposed model to ARIMA and ARIMAX, and the RF model outperforms with MAPE of approximately 20% to the ARIMA model, with MAPE reaching 30%.” should be corrected as “They compared the proposed model to ARIMA and ARIMAX, and the RF model with the MAPE of 20% outperforms the ARIMA model with the MAPE of 30%.” In addition, the y-axis title is missing in the first graph of Fig. 1.
